# Impact of Dietary Supplementation of Spice Extracts on Growth Performance, Nutrient Digestibility and Antioxidant Response in Broiler Chickens

**DOI:** 10.3390/ani13020250

**Published:** 2023-01-10

**Authors:** Javier Herrero-Encinas, Almudena Huerta, Marta Blanch, José Javier Pastor, Sofia Morais, David Menoyo

**Affiliations:** 1 Departamento de Producción Agraria, Universidad Politécnica de Madrid, ETS Ingeniería Agronómica, Alimentaria y de los Biosistemas, 28040 Madrid, Spain; 2 Lucta S. A., Innovation Division, Animal Science Unit, UAB Research Park, Edifici Eureka, 08193 Bellatrra, Spain

**Keywords:** broiler chicken, phytogenic additive, spices, performance, nutrient digestibility

## Abstract

**Simple Summary:**

Capsaicin is a bioactive component that is obtained mainly from chili peppers and is a well-recognized antimicrobial agent, modulator of the immune response, and enhancer of nutrient digestibility, with a good potential to improve productivity in farm animals. This study explored the mechanism of action of a mixture of extracts containing capsaicin as the main component, together with black pepper and ginger, which was previously shown to exert positive effects on broiler chicken growth performance. Effects on nutrient digestibility, digestive enzyme activity, and plasma, jejunum and liver antioxidant response were examined. Results showed an enhancement of growth parameters, mainly in early stages; improvement of dry matter, crude protein and energy apparent ileal digestibility; and also effects on antioxidant enzyme activity in plasma and liver.

**Abstract:**

This study aimed to investigate the effects of supplementing broiler chicken diets with an encapsulated product based on capsicum and other spice (black pepper and ginger) extracts on growth performance, nutrient digestibility, digestive enzyme activity and antioxidant response. To this end, 480 1-day-old male chicks were randomly assigned to two experimental treatments (12 pens/treatment; 20 birds/pen). Dietary treatments included a basal diet with no additives (CONTROL) and a basal diet supplemented with 250 ppm of the spice additive (SPICY; Lucta S.A., Spain). Supplementation of SPICY increased body weight (*p* < 0.05) compared with CONTROL at 7 d of age and improved (*p* < 0.01) ADG from 0 to 7 d of age. The apparent ileal digestibility of dry matter, gross energy and crude protein was higher (*p* < 0.05) in birds fed the SPICY diet compared with the CONTROL diet. Birds fed SPICY showed lower (*p* < 0.05) plasma catalase (CAT) activity, and the hepatic gene expression of CAT and Nrf2 was down-regulated (*p* < 0.05) compared with the CONTROL. In conclusion, the inclusion of 250 ppm of SPICY in broiler diets improved growth performance at 7 d of age and positively affected nutrient digestibility and antioxidant response.

## 1. Introduction

Spices are substances derived from non-leaf parts of plants, including seeds, fruits, barks and roots, with intensive taste or smell, most commonly known by their widespread culinary use as food condiments. However, owing to their high concentration in bioactive substances, they are also used as nutraceuticals and can be considered as phytogenic feed additives with demonstrated beneficial effects on poultry growth performance and health [1,2,3].

The positive effects of spices in farmed animals, including poultry, might be partly attributed to an increased feed palatability associated with the presence of volatile compounds, flavors and colors that can enhance feed intake and growth efficiency [4,5]. However, other important properties of spices are associated with the presence of bioactive components with positive effects on lipid metabolism, stimulation of digestion, antioxidant effects and anti-inflammatory properties [6,7,8]. Capsaicin, for example, the principal active compound in capsicum oleoresin derived from red pepper (*Capsicum* spp.), has been described as possessing analgesic, antioxidant, and antimicrobial effects [9]. Black pepper (*Piper nigrum* L.), another well-known spice that is rich in piperine and other bioactive substances, including piperic acid, piperlonguminine or pellitorine, displayed strong antioxidant, anti-inflammatory and antimicrobial properties [10]. Likewise, ginger (*Zingiber officinale* Rosc.), a rhizome containing a high proportion of bioactive compounds, including gingerols, paradols or zingerones, displayed positive immune and antioxidant effects when administered to poultry feed [11]. Furthermore, the combination of certain spices could have a synergistic effect. For example, Platel et al. [12] showed that the diverse spices combination in rat diets enhanced digestive processes by increasing pancreatic enzyme activity and bile secretions. Also, the bioavailability of some phytochemicals can be enhanced by the piperine supplementation because of changes in the dynamics of lipid membrane and alterations in intestine enzymes exerted by this alkaloid present in black pepper [13].

Previous studies of broiler chickens have demonstrated that the inclusion of capsaicin, black pepper and ginger in feed, separately or in mixture, is able to improve broiler chicken growth performance, improve digestive enzyme activity, and modulate gut microbiota and oxidative status [14,15,16,17,18]. In this regard, Menoyo et al. [19] have demonstrated a significant improvement in broiler chicken growth performance and feed efficiency when performing a meta-analysis of eight trials using a blend of the three above-mentioned pungent spices. Additionally, Ipharraguerre et al. [20] showed an enhancement of ether extract fecal digestibility in broiler chickens supplemented with the same mixture of extracts. This improvement was even higher in diets with saturated fat from animal origin. Nevertheless, the mechanism(s) of action of this blend of spices explaining beneficial effects in broiler performance and digestibility has not been completely elucidated and deserves to be further examined. Thus, the objective of the present study was to evaluate the effects of the blend, when added to broiler feeds, on the animal’s growth performance, nutrient digestibility, digestive enzyme activity and antioxidant enzyme activity.

## 2. Materials and Methods

### 2.1. Housing and Experimental Animals

All animal care and experimental procedures were approved by the Ethics Committee of the Universidad Politécnica de Madrid and are in compliance with the Spanish Guidelines for the Care and Use of Animals in Research [21].

A feeding trial was carried out at the Universidad Politécnica de Madrid experimental facilities (Department of Agricultural Production and E.T.S.I. Agronómica, Alimentaria y de Biosistemas). A total of 480 1-day-old male chicks (Cobb 500; 42.6 ± 0.06 g) were obtained from a commercial hatchery (Incubadora Uvesa, Tudela, Navarra, Spain). Broiler chickens were randomly distributed among 24 floor pens (12 pens/treatment, 1 × 1.5 m) with 20 birds each. Floor pens were equipped with a hopper feeder, bell drinker and wood shaving bedding. The facility temperature was set at 33 ± 1 °C at the start of the trial and gradually decreased to 23 ± 1 °C by 21 d of age. Humidity and ventilation were automatically controlled. A photoperiod of 24L:0D was set during the first 7 d of age and 18L:6D until the end of the experiment at 21 days of age.

### 2.2. Diets and Experimental Design

The design was completely randomized with two treatments: a CONTROL with no additives (CONTROL) and an experimental diet obtained by adding on top of the basal diet 250 ppm of an encapsulated product (SPICY, Lucta, S.A., Madrid, Spain) containing capsicum as the main component, blended with black pepper and ginger extracts. Diets were formulated to have the same nutritive value according to FEDNA [22] and were manufactured at Instituto de Ciencia y Tecnología Animal of the Universitat Politécnica de Valencia. The composition of the basal diet is shown in Table 1. Titanium dioxide was added to the feed, at 5 g/kg of feed, as a marker for apparent ileal digestibility (AID) determination. Broiler chickens were fed their respective experimental diets ad libitum in crumbles from 1 to 21 days of age.

### 2.3. Productive Traits and Sampling

To calculate the average daily gain (ADG), average daily feed intake (ADFI), and feed conversion ratio (FCR), body weight and feed consumption were determined by pen at 7, 14 and 20 d of age. Mortality was recorded and weighed as occurred. At the end of the experiment at 21 days of age, birds were euthanized by inhalation of CO_2_. One bird per pen was randomly selected and sampled to analyze the activity of pancreatic enzymes, liver and jejunum gene expression, plasma α-tocopherol concentration and antioxidant enzyme activity. For pancreatic enzyme activity, 1 g of pancreas was collected and stored at −80 °C. Blood samples were collected immediately postmortem via cardiac puncture using sterile syringes and needles. To obtain the plasma, blood was collected into tubes containing EDTA and aprotinin (BD Vacutainer, Plymouth, UK), centrifuged at 2000× *g* for 10 min and stored at −80 °C for further analysis of α-tocopherol, glutathione peroxidase, glutathione S-transferase, superoxide dismutase, and catalase plasma concentration. For gene expression analysis, approximately 200 mg of jejunum mucosal scrapings and 500 mg of liver were taken in RNA later (Invitrogen, Carlsbad, CA) following the manufacturer’s instructions, and further stored at −80 °C.

### 2.4. Apparent Ileal Digestibility Analysis

At 21 days of age, eight birds per pen were randomly selected and euthanized for the collection of ileal digesta content. Ileal digesta (Meckel’s diverticulum to ileocecal junction) was squeezed into 120 mL cups and immediately stored in dry ice. Then, samples were frozen at −80 °C, lyophilized, grounded (0.50 mm of particle size), and stored in air-tight cups until further analysis. Apparent ileal digestibility of dry matter, gross energy, ether extract, and crude protein were determined using titanium dioxide in feed (5 g TiO_2_/kg of feed) as an inert marker and calculated using the following equation:

AID (%) = 100 × [1 − ((TiO_2_ diet)/(TiO_2_ digesta)) × ((Nutrient diet)/(Nutrient digesta))]

where TiO_2_ digesta corresponded to the ileal TiO_2_ concentration, TiO_2_ diet is the concentration of TiO_2_ in feed, and Nutrient digesta and diet are the values of the dry matter (DM), gross energy (GE), ether extract (EE) or crude protein (CP) in the ileal content and feed, respectively. 

### 2.5. Chemical Analysis

Diets and ileal digesta samples were analyzed following the standard methods of AOAC [23] for DM (934.01), EE (920.39), and CP (968.06). The GE was analyzed using a 6400 automatic isoperibol oxygen bomb calorimeter (Parr Instruments, Moline, IA, USA). Additionally, diets and digesta were analyzed for TiO_2_ concentrations in triplicate by the method described by Short et al. [24].

### 2.6. Enzyme Activity and Plasma Analysis 

Pancreatic enzyme activities were determined using the respective commercial kit. Specifically, amylase activity (Sigma Kit MAK009; Sigma, St. Louis, MO, USA), trypsin activity (Sigma Kit MAK290; Sigma, St. Louis, MO, USA), and lipase activity (Sigma Kit MAK046; Sigma, St. Louis, MO, USA) were performed according to the instructions of the manufacturer. 

Plasma enzyme activities were determined using commercial kits and following the manufacturer’s instructions, particularly glutathione peroxidase activity (GPx, Cayman Chemical Kit catalogue no. 703102; Cayman Chemical, Ann Arbor, MI, USA), glutathione S-transferase activity (GST, Cayman Chemical Kit catalogue no. 703302; Cayman Chemical, Ann Arbor, MI, USA), superoxide dismutase activity (SOD, Cayman Chemical Kit catalogue no. 706002; Cayman Chemical, Ann Arbor, MI, USA), and catalase activity (CAT, Cayman Chemical Kit catalogue no. 707002; Cayman Chemical, Ann Arbor, MI, USA). 

The concentration of α-tocopherol in plasma was determined using reverse-phase HPLC as described in Rey et al. [25].

### 2.7. Gene Expression Analysis

Total RNA was extracted from approximately 30 mg of liver and jejunum as previously described in Herrero et al. [26]. Extracted RNA yield and quality were measured by absorbance at wavelengths of 260 and 280 nm. Starting from around 2400 ng of extracted RNA, the first DNA strand was obtained by its reverse transcription, performed by SuperScript VILO Master Mix (Invitrogen, Life Technologies, Carlsbad, CA, USA). Quantitative real-time PCR analysis was performed in a 7300 Real Time PCR System (Applied Biosystems, Foster City, CA). Primers and PCR conditions for the chicken ubiquitin (UB, housekeeping), actin beta (ACTB, housekeeping), catalase (CAT), glutathione peroxidase 1 (GPx1), superoxide dismutase 1 (SOD1), and nuclear factor erythroid 2-related factor 2 (Nrf2) were obtained from the literature (Table 2). Specific product amplification was checked by the melting curve analysis. Gene qRT-PCR efficiencies were evaluated by generating standard curves using cDNA from a pool of samples and calculated according to the equation E = 10 (−1/slope). Samples were analyzed in duplicate using the right amount of each primer, ultra-purified water, and SYBR Green Master Mix (Applied Biosystems, Life Technologies, Carlsbad, CA, USA). 

### 2.8. Statistical Analysis

All statistical analyses were made using SAS [31] (release 9.2; SAS Institute, Cary, NC, USA) with experimental diets as a fixed effect. Normality distribution was checked using Shapiro–Wilk and Kolmogorov–Smirnov tests, and Levene’s test was used to confirm the homogeneity of variance of data. Growth performance, apparent ileal digestibility, pancreatic enzyme activities, plasma alpha-tocopherol, and antioxidant enzyme activities data were analyzed by Student’s *t*-test. Differences were declared significant at probability level *p* < 0.05, tendencies were significant at probability level 0.05 < *p* < 0.10, and results were presented as mean ± SEM. Trypsin pancreatic activity was square root transformed before being statistically analyzed to satisfy population normativity and variance homogeneity assumptions. 

## 3. Results

### 3.1. Animal Performance and Mortality

Growth performance data are shown in Table 3. Body weight was significantly lower (*p* < 0.05) for birds in the CONTROL group compared with those in the SPICY group at 7 d of age, but there was no difference (*p* > 0.05) in BW at 14 and 20 d of age. Furthermore, ADG was lower (*p* < 0.05) in CONTROL birds compared with SPICY birds during the period from 0 to 7 d of age, with birds displaying higher (*p* < 0.05) FCR in CONTROL treatment than in SPICY treatment. No differences (*p* > 0.05) were observed in ADG, ADFI, and FCR among treatments from 7 to 14 and 0 to 20 d of age. Mortality was less than 5% and unrelated to treatment (data not shown).

### 3.2. Apparent Ileal Digestibility

Results of AID are shown in Table 4. The AID of dry matter, gross energy, and crude protein were significantly (*p* < 0.05) higher in SPICY birds compared with CONTROL birds at 21 d of age. By contrast, no differences (*p* > 0.05) were observed in AID of ether extract among the experimental treatments. 

### 3.3. Pancreatic Enzyme Activity

Pancreatic enzyme activity data are shown in Table 5. The activity of amylase tended (*p* = 0.063) to be higher in the pancreas of SPICY birds compared with CONTROL birds at 21 d of age. Additionally, no significant differences (*p* > 0.05) were observed in trypsin and lipase activity among experimental treatments.

### 3.4. Plasma Alpha Tocopherol Concentration and Antioxidant Enzyme Activity

Results of the concentration of α-tocopherol and antioxidant enzyme activity in plasma are shown in Table 6. The concentration of α-tocopherol was not (*p* > 0.05) affected by dietary experimental treatments. Furthermore, no significant differences (*p* > 0.05) were observed in the activity of GPx, GST, and SOD among experimental treatments. However, CAT activity was significantly (*p* < 0.05) lower in plasma of birds fed the SPICY diet compared with CONTROL at 21 days of age. 

### 3.5. Gene Expression

**Figure 1:**
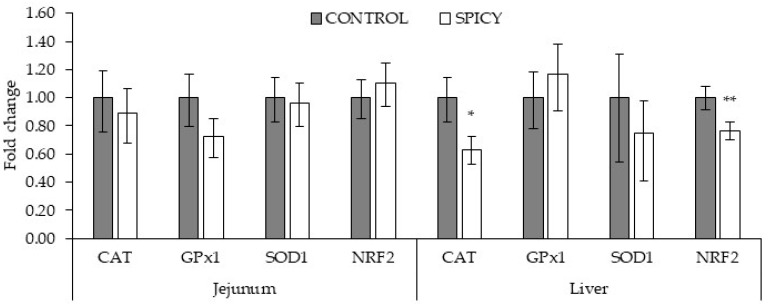
Effect of experimental diets on broiler chicken jejunum and liver gene expression at 21 d of age. CAT, catalase; GPx1, glutathione peroxidase 1; SOD1, superoxide dismutase 1; Nrf2, nuclear factor erythroid 2-related factor 2. Gene expression values are indicated as fold changes relative to the mRNA levels in CONTROL set to be 1.0. Bars indicate the 95% confidence interval (fold change up-fold change low) (*n* = 12; *: *p* < 0.05; **: *p* < 0.01).

No differences (*p* > 0.05) were observed in jejunum gene expression of CAT, GPx1, SOD1, and Nrf2 among experimental treatments at 21 d of age (Figure 1). Additionally, the expression of GPx1 and SOD1 in the liver was not affected (*p* > 0.05) by dietary treatment. However, liver gene expression of CAT and Nrf2 was significantly (*p* < 0.05) lower in the SPICY treatment compared with the CONTROL.

## 4. Discussion

A recent meta-analysis of eight studies testing diet supplementation with the blend of SPICY extracts demonstrated significant enhancement of performance in broiler chickens [19]. In the present study, supplementing broiler diets with 250 ppm of SPICY during the first week improved the BW and ADG and tended to increase FCR compared with a no supplemented CONTROL. However, despite BW being numerically higher in the following periods (973 vs. 982 g for CONTROL and SPICY, respectively, at 20 d of age), it did not reach statistical significance. In the global period, from 1 to 20 d of age, no significant differences were observed in the FCR. These results are partially inconsistent with the previous meta-analysis [19], which showed a tendency to improve ADG but a significant improvement of FCR with SPICY in the starter phase (1 to 21 d of age). Thiamhirunsopit et al. [32] observed a growth enhancement with 20 to 30 mg/kg of capsaicin supplementation in broiler chickens under high stocking density conditions. In addition, Bravo et al. [33] and Pirgozliev et al. [34] reported improved performance and energy utilization for growth in broilers fed a mixture of capsaicin, carvacrol and cinnamaldehyde under poor hygienic conditions. These authors suggested that the efficiency of this capsaicin blend is influenced by the rearing conditions, being more efficient under adverse situations such as poor hygienic conditions. In the previously mentioned meta-analysis performed with SPICY studies, some of the trials were performed under commercial conditions, so it is plausible that the lack of clear effects on FCR in the global period in the present study might be related to the optimal rearing conditions [19]. Studies focused on the effects of black pepper and ginger extracts in broiler chicken diets are available in the literature. Abou-Elkhair et al. [35] showed an improvement in body weight gain and gain-to-feed ratio of broiler chickens fed diets supplemented with 0.5% of black pepper compared with control. However, Cardoso et al. [36] were unable to detect beneficial effects of diet supplemented with black pepper in body weight of broiler chickens. Furthermore, the supplementation with ginger root powder or extracts in broiler chicken diets have shown contradictory results in growth performance [37,38]. Discrepant results between different studies are not completely unexpected considering that the effectiveness of dietary spices, as in most phytogenic additives, depends on many different factors related to the botanical itself, animal management (Abdelli et al.) [39] and the use of single or combined spices. 

The supplementation with SPICY improved the AID of dry matter, gross energy, and crude protein compared with the non-supplemented CONTROL diet. These results are in line with those of Liu et al. [15], who observed better AMEn and higher digestibility of organic matter and crude protein in broiler chickens supplemented with 80 mg/kg of a capsicum extract. However, Thiamhirunsopit et al. [32] showed no significant effect of 20–30 mg/kg of capsaicin supplementation on ileal nutrient digestibility at 21 and 41 d of age. A previous study with SPICY observed increased fecal digestibility of fat when comparing lard and soybean oil in broiler chickens at 21 d of age [20]. The present study does not indicate a significant effect on fat digestibility, which is consistent with the study by Thiamhirunsopit et al. [32]. Other authors showed that the beneficial effect of capsaicin on nutrient digestibility is due to the enhancement of enzyme activities in the gastrointestinal tract and the enhancement of bile secretion [6]. Studies conducted by Prakash and Srinivasan [40] and Platel and Srinivasan [41] reported an increment of trypsin, lipase, amylase, and chymotrypsin activity in the pancreas of rats fed diets supplemented with capsaicin, ginger, curcumin or piperine. In the previously mentioned study with broiler chickens [15], where the dietary inclusion of 80 mg/kg of a capsicum extract resulted in higher digestibility of organic matter, crude protein, and AMEn at 42 d of age, results were associated with a significantly higher activity of trypsin and lipase. In addition, Li et al. [42] observed higher amylase, lipase, and trypsin activities on jejunal and ileal content with the supplementation of 2–6 mg/kg of capsaicin in broiler chicken diets at 21 and 42 d of age. Furthermore, Long et al. [43] suggested that dietary supplementation of 80 mg/kg of a capsicum extract (containing 2% of capsaicin) in weaned pigs improved the apparent total tract digestibility of dry matter, crude protein, gross energy, and organic matter through the enhancement of intestine maturation and enzyme activity in the ileal and jejunal mucosa. On the other hand, other pungent spices, including raw black pepper and ginger or derived extracts, which are also components of the SPICY supplement, albeit at lower levels than capsicum, have also been proven to have beneficial effects in nutrient digestibility when added to broiler diets. Recently, Al-Khalaifah et al. [18] observed an enhancement of dry matter, crude protein, crude fiber and ether extract utilization in broilers fed diets supplemented with 5 to 15 g/kg of ginger powder. Similarly, as previously noted with capsaicin, the beneficial effects of ginger supplementation in terms of nutrient digestibility seem to be related to the higher activity of digestive enzymes such as protease, lipase, and maltase [44,45]. Furthermore, piperine, the major bioactive principle of black pepper, has shown digestive stimulatory effects through the increase of lipase activity, gastric acid secretion, bile flow and pepsin secretion in rodents and humans [46]. However, Oso et al. [47] observed no significant differences in crude fat and crude protein AID in broiler chickens fed diets supplemented with a blend of spices that contained piperine compared with a control diet with antibiotics. Nevertheless, in spite of the well-documented effect of these spices in enhancing digestive enzyme activity, the results of the present study showed no significant effect of SPICY on trypsin and lipase activity, although the supplemented diet tended to increase by 46% the activity of pancreatic amylase compared with the CONTROL diet. This result is puzzling, especially considering that digestibility of most fractions assessed was positively affected. However, other reported effects of spices that could underline an enhancement of digestive function have not been measured in the present study and could have contributed to the results. These potential effects include increasing salivary and gastric secretion, secretion of bile (through increased turnover of cholesterol into bile salts) and stimulation of enzymes of the intestinal mucosa (brush border proteolytic enzymes and peptidases) [4].

Several phytochemical spices improve health by boosting antioxidant defenses and reducing oxidative stress [48]. In intensive broiler chicken production, birds are exposed to several stressors, including poor environmental conditions, pathogens, and unbalanced diets, which can alter body homeostasis and trigger oxidative stress, leading to decreased performance [49]. The secretion of antioxidant enzymes such as SOD, GPx, GSP or CAT, and other antioxidant substances including α-tocopherol and ascorbic acid, counteract and protect from the oxidative damage produced by reactive oxygen species (ROS) [50]. Recent data indicate that phytochemical protective effects in poultry are mediated, among others, by antioxidant enzyme function through Nrf2 activation [51]. In the present study, the blend of capsicum, black pepper and ginger extracts supplemented to the diet exerted a lower CAT activity in plasma compared with CONTROL, but no significant changes in plasma GPx, GSP, and SOD activities. In addition, gene expression of CAT, GPx1, SOD1 and Nrf2 in the jejunum, and GPx1 and SOD1 in the liver, were not affected by SPICY supplementation. However, supplementation of SPICY in diets caused a downregulation in CAT and Nrf2 expression in the liver. These results are somehow unexpected as Liu et al. [15] observed that supplementation with 80 mg/kg of capsicum increased the serum activity of GPx and SOD and liver CAT activity in broiler chickens. Similarly, diet supplementation with ginger powder led to an increment of hepatic SOD and CAT activity in broiler chickens [18]. By contrast, Mueller et al. [52] showed that the inclusion of broccoli extract and various essential oils (from turmeric, oregano, thyme, and rosemary) increased the expression of antioxidant enzymes in jejunum but reduced them and Nrf2 expression in the liver. According to the authors, the increased antioxidant function at the intestinal level with the phytogenics protects peripheral organs like the liver from oxidative stress, making the induction of antioxidant enzymes dispensable [52]. In the present study the concentration of plasma α-tocopherol was not significantly different but numerically higher (around 13%) in birds fed the SPICY diet (6.09 vs. 6.90 µg/mL for CONTROL and SPICY, respectively). Therefore, it is plausive that the reduced CAT activity in plasma and the downregulation of CAT and Nrf2 genes in the liver of the SPICY fed chicks might be indicative of a lower need of activation of antioxidant defense enzymes in peripheral tissues. This hypothesis merits future studies. 

## 5. Conclusions

Consistent with previous studies investigating the effects of a SPICY mixture, the present study shows that the dietary inclusion of 250 ppm of encapsulated product based on capsicum and other spice (black pepper and ginger) extracts positively affects growth performance in broiler chickens during the first week of life. Positive effects on weight gain might be attributable, at least partly, to an improvement in nutrient digestibility, and probably by enhancing amylase activity. Additionally, results suggest that SPICY supplementation could enhance the oxidative stress defensive system of broiler chickens, making the induction of CAT dispensable in plasma and liver.

## Figures and Tables

**Table 1:** Ingredients calculated and analyzed chemical composition of the basal diet (%, as fed basis, unless otherwise indicated).

Item	Basal Diet
Maize	50.88
Soybean meal (46.5% CP)	39.97
Lard	4.00
Dicalcium phosphate	1.97
Calcium carbonate	1.11
Titanium dioxide	0.50
Vitamins and Minerals ^1^	0.50
Sodium bicarbonate	0.32
DL-methionine	0.30
Sodium chloride	0.23
L-lysine HCl	0.16
L-threonine	0.06
Calculated composition	
AMEn ^2^ (kcal/kg)	2950
Crude fiber	2.91
Crude protein	22.7
Lysine	1.22
Methionine	0.60
Methionine + cysteine	0.90
Threonine	0.79
Tryptophan	0.24
Calcium	1.02
Digestible phosphorus	0.45
Sodium	0.19
Analyzed composition (% DM)	
Dry matter	89.3
TiO_2_	0.46
Crude protein	23.8
Ether extract	6.50
Gross energy (kcal/kg)	4035

^1^ Providing the following (per kilogram of diet): vitamin A (trans retinyl acetate), 7.500 IU; vitamin D3 (cholecalciferol), 2.000 IU; vitamin E (all-rac-tocopherol acetate), 9 mg; vitamin K (bisulfate menadione complex), 2 mg; riboflavin, 5.5 mg; pantothenic acid (D-calcium pantothenate), 9 mg; nicotinic acid, 25 mg; pyridoxine (pyridoxine·HCl), 1.85 mg; vitamin B12 (cyanocobalamine), 12.5 μg; D-biotin, 0.10 mg; folic acid, 0.5 mg; Betaine-HCl, 175 mg; Se (Na_2_SeO_3_), 0.2 mg; I (KI), 1.8 mg; Cu (CuSO_4_·H_2_O), 6.25 mg; Fe (FeSO_4_·H_2_0), 30 mg; Zn (ZnO), 52 mg; Mn (MnSO_4_·H_2_O), 80 mg; BHT, 0.16 mg. ^2^ AMEn, apparent metabolizable energy.

**Table 2:** Genes, forward and reverse primers, for gene expression analysis by quantitative real-time PCR.

Gene ^1^	5′-Primer Sequence Forward-3′	5′-Primer Sequence Reverse-3′	Reference
UB	GGGATGCAGATCTTCGTGAAA	CTTGCCAGCAAAGATCAACCTT	[27]
ACTB	GTGATGGACTCTGGTGATGG	TGGTGAAGCTGTAGCCTCTC	[28]
CAT	GAGATGGTGAGGGCAGTTATT	GCCAATGTATGAGGAGGTTAGT	[29]
GPx1	CCACTTCGAGACCATCAAACT	GGTGCGGGCTTTCCTTTA	[29]
SOD1	TGGCTTCCATGTGCATGAAT	AGCACCTGCGCTGGTACAC	[30]
Nrf2	CAGAAGCTTTCCCGTTCATAGA	TGGGTGGCTGAGTTTGATTAG	[29]

^1^ UB, ubiquitin; ACTB, actin beta; CAT, catalase; GPx1, glutathione peroxidase 1; SOD1, superoxide dismutase 1; Nrf2, nuclear factor erythroid 2-related factor 2.

**Table 3:** Effect of experimental diets on broiler chicken growth performance from 0 to 20 d of age ^1^.

Item ^2^	CONTROL	SPICY	*p*-Value
BW (g/bird)			
0 d	42.5 ± 0.17	42.6 ± 0.18	0.55
7 d	173 ± 1.36	177 ± 0.90	0.007
14 d	494 ± 3.20	501 ± 3.89	0.15
20 d	973 ± 4.64	982 ± 10.0	0.43
0’7 d			
ADG (g/bird/d)	18.6 ± 0.18	19.3 ± 0.12	0.005
ADFI (g/bird/d)	17.8 ± 0.19	17.9 ± 0.14	0.87
FCR (g/g)	0.94 ± 0.004	0.93 ± 0.005	0.054
7’14 d			
ADG (g/bird/d)	46.1 ± 0.19	46.3 ± 0.40	0.61
ADFI (g/bird/d)	52.6 ± 0.36	53.4 ± 0.55	0.22
FCR (g/g)	1.15 ± 0.006	1.15 ± 0.004	0.59
14’20 d			
ADG (g/bird/d)	79.9 ± 0.69	80.3 ± 1.15	0.76
ADFI (g/bird/d)	92.5 ± 0.78	93.6 ± 1.11	0.44
FCR (g/g)	1.16 ± 0.005	1.17 ± 0.012	0.66
0’20 d			
ADG (g/bird/d)	46.5 ± 0.23	47.0 ± 0.50	0.43
ADFI (g/bird/d)	52.5 ± 0.30	53.0 ± 0.39	0.36
FCR (g/g)	1.13 ± 0.004	1.13 ± 0.006	0.99

^1^ Data are means ± standard error (*n* = 12 replicates with 20 birds each). ^2^ BW, body weight; ADG, average daily gain; ADFI, average daily feed intake; FCR, feed conversion ratio.

**Table 4:** Effect of experimental diets fed to broiler chickens on nutrient apparent ileal digestibility (%) at 21 d of age ^1^.

Item	CONTROL	SPICY	*p*-Value
Dry matter	62.9 ± 1.15	66.3 ± 0.54	0.016
Gross energy	67.2 ± 1.06	70.2 ± 0.50	0.022
Ether extract	80.8 ± 1.10	82.1 ± 0.93	0.39
Crude protein	79.2 ± 0.52	80.9 ± 0.54	0.031

^1^ Data are means ± standard error (n = 12 replicates with 20 birds each).

**Table 5:** Effects of experimental diets on the activity of pancreatic enzymes in broilers at 21 d of age ^1^.

Item	CONTROL	SPICY	*p*-Value
Amylase ^2^ (U/mL)	174 ± 18	254 ± 35	0.063
Trypsin ^3^ (mU/mL)	411 ± 47	415 ± 76	0.96
Lipase ^4^ (mU/mL)	0.58 ± 0.14	0.66 ± 0.14	0.68

^1^ Data are means ± standard error (n = 12 replicates). ^2^ One unit of amylase is the amount of amylase that cleaves ethylidene-pNP-G7 to generate 1.0 mmole of p-nitrophenol per minute at 25 °C. ^3^ One unit of trypsin is defined as the amount of trypsin that cleaves the substrate, yielding 1.0 µmole of p-NA per minute at 25 °C. The *p*-values of trypsin are from square root data transformation analysis. ^4^ One unit of lipase is defined as the amount of enzyme that will generate 1.0 μmol of glycerol from triglycerides per minute at 37 °C.

**Table 6:** Effects of experimental diets on plasma alpha-tocopherol and antioxidant activity of enzymes in broilers at 21 days of age ^1^.

Item	CONTROL	SPICY	*p*-Value
α-tocopherol (µg/mL)	6.09 ± 0.37	6.90 ± 0.40	0.16
GPx (mU/mL) ^2^	3283 ± 132	3329 ± 122	0.80
GST (mU/mL) ^3^	27.4 ± 1.17	30.4 ± 1.51	0.13
SOD (%) ^4^	43.5 ± 7.07	39.5 ± 3.61	0.59
CAT (U/mL) ^5^	1.54 ± 0.070	1.30 ± 0.080	0.041

^1^ Data are means ± standard error (*n* = 12 replicates). ^2^ One unit of glutathione peroxidase is defined as the amount of enzyme that will cause the oxidation of 1.0 nmol of NADPH to NADP per minute at 25 °C. ^3^ One unit of glutathione S-transferase will conjugate 1.0 nmol of 1-chloro-2,4-dinitrobenzene with reduced glutathione per minute at 25 °C. ^4^ One unit of superoxide dismutase is defined as the amount of enzyme needed to exhibit 50% dismutation of the superoxide radical measured in change in absorbance per minute at 25 °C and pH 8.0. ^5^ One unit of catalase is the amount of enzyme that will cause the formation of 1.0 nmol of formaldehyde per minute at 25 °C.

## Data Availability

The data presented in this study are available on request from the corresponding author.
